# Supplementary material for: Fast simulation of identity-by-descent segments
Source: bioRxiv. 2025 Jan 7:2024.12.13.628449. Preprint. [Version 2] doi: 10.1101/2024.12.13.628449 (PMC11741331; doi:10.1101/2024.12.13.628449)
Supplement: Supplement 1 [file NIHPP2024.12.13.628449v2-supplement-1.pdf]

bioRxiv preprint doi: https://doi.org/10.1101/2024.12.13.628449; this version posted January 7, 2025. The copyright holder for this preprint (which was not certified by peer review) is the author/funder, who has granted bioRxiv a license to display the preprint in perpetuity. It is made available under aCC-BY 4.0 International license.

709 # Supplementary figures

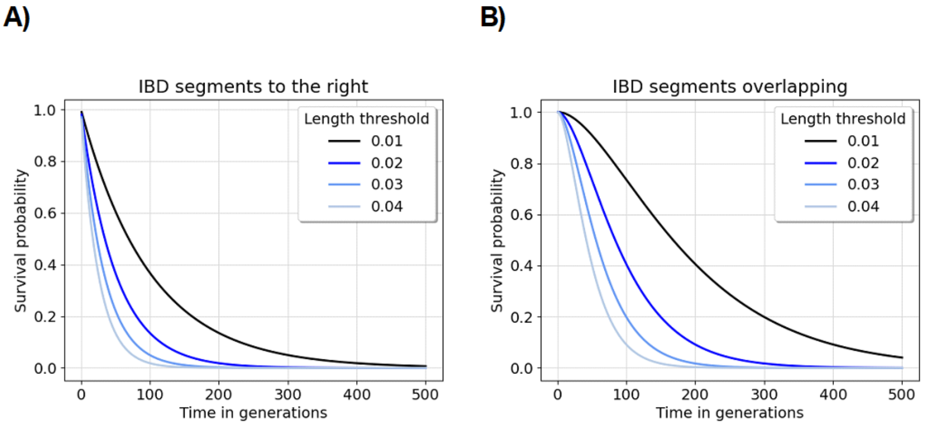

Fig. S1 The upper tail probabilities of Gamma random variables. Subplots A) and B) show the survival probabilities for shape parameters 1 and 2, respectively. The rate of the random variables is the coalescent time in generations (x-axis). The survival probability (y-axis) comes from Equations 6 and 7. The length thresholds are denoted by different colors and line styles, defined in the legend.

bioRxiv preprint doi: https://doi.org/10.1101/2024.12.13.628449; this version posted January 7, 2025. The copyright holder for this preprint (which was not certified by peer review) is the author/funder, who has granted bioRxiv a license to display the preprint in perpetuity. It is made available under aCC-BY 4.0 International license.

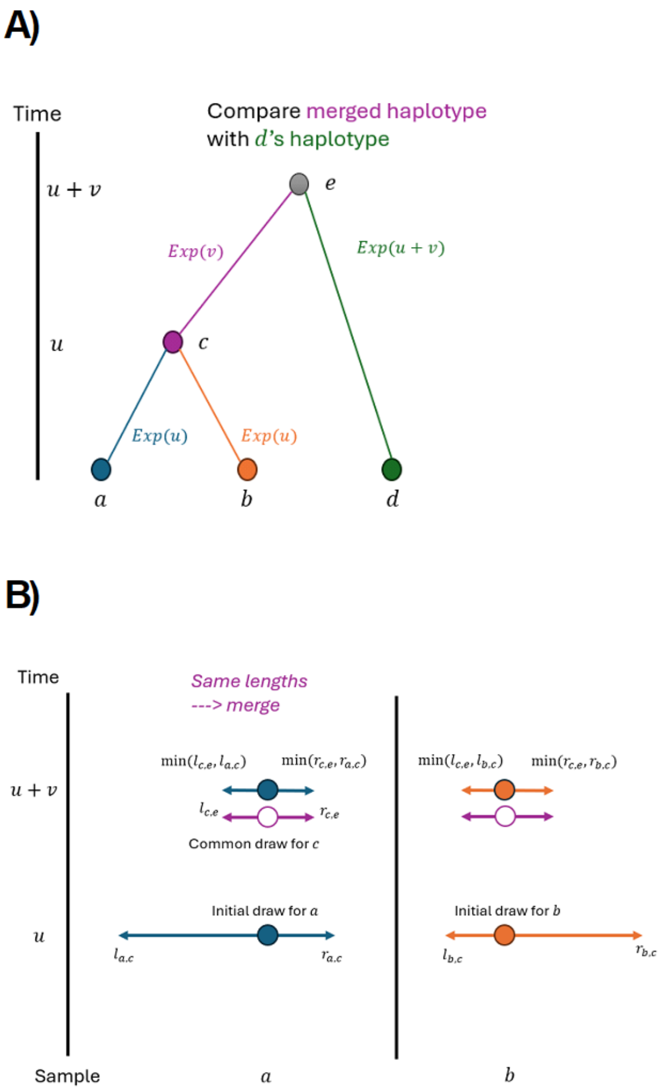

Fig. S2 Illustration of merging haplotypes. A) We draw recombination endpoints to the left and right of the focal location from Exponential(u) for both sample haplotypes a and b at coalescent time u. We draw recombination endpoints to the left and right of the focal location from Exponential(v) for the common ancestor c of a and b at coalescent time $u + v$. Colors denote branch lengths and recombination endpoints corresponding to a given haplotype. $l_{a,c}$ and $r_{a,c}$ denote the endpoints for a drawn to the left and right of the focal location at time u (lowercase denotes observation of random variables). B) We compute minimums of lengths drawn for c and a and c and b, respectively. We merge the sample haplotypes a and b in future calculations because the minimum lengths are both the recombination endpoints drawn from the common ancestor c. When comparing recombination endpoints at time $u + v$ with those of the haplotype d, we make one comparisons. The haplotypes remain longer than the detection threshold w Morgans.

bioRxiv preprint doi: https://doi.org/10.1101/2024.12.13.628449; this version posted January 7, 2025. The copyright holder for this preprint (which was not certified by peer review) is the author/funder, who has granted bioRxiv a license to display the preprint in perpetuity. It is made available under aCC-BY 4.0 International license.

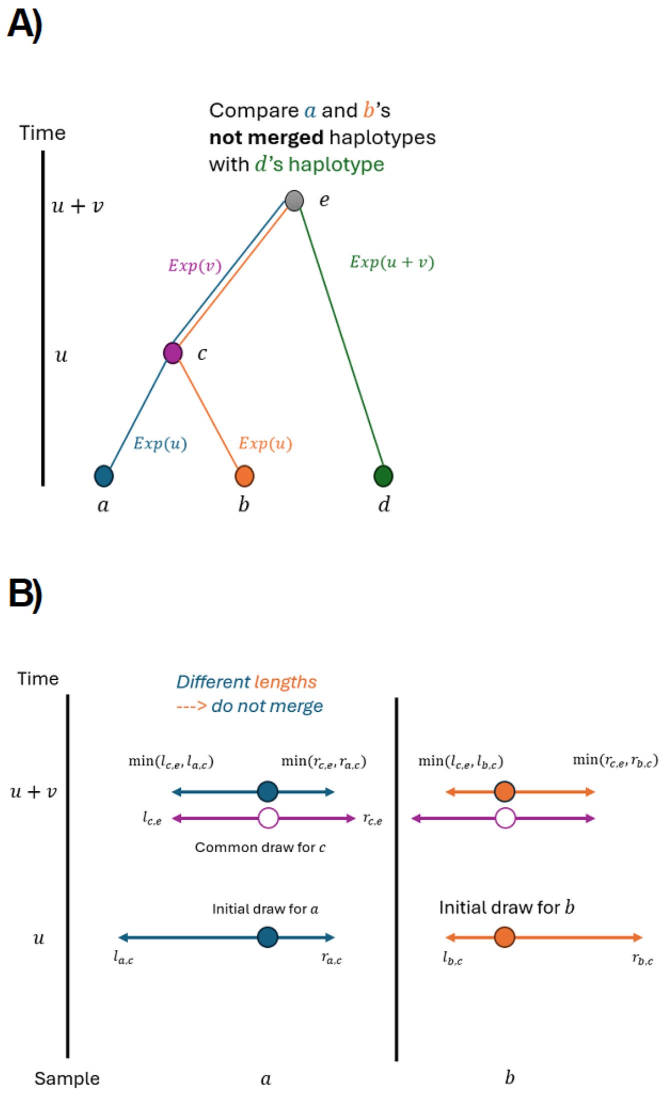

Fig. S3 Illustration of ***not*** merging haplotypes. A) We draw recombination endpoints to the left and right of the focal location from Exponential(u) for both sample haplotypes a and b at coalescent time u. We draw recombination endpoints to the left and right of the focal location from Exponential(v) for the common ancestor c of a and b at coalescent time $u + v$. Colors denote branch lengths and recombination endpoints corresponding to a given haplotype. $l_{a,c}$ and $r_{a,c}$ denote the endpoints for a drawn to the left and right of the focal location at time u (lowercase denotes observation of random variables). B) We compute minimums of lengths drawn for c and a and c and b, respectively. We ***do not*** merge the sample haplotypes a and b in future calculations because the minimum lengths are ***not*** both the recombination endpoints drawn from the common ancestor c. When comparing recombination endpoints at time $u+v$ with those of the haplotype d, we make ***two*** comparisons. The haplotypes remain longer than the detection threshold w Morgans.

bioRxiv preprint doi: https://doi.org/10.1101/2024.12.13.628449; this version posted January 7, 2025. The copyright holder for this preprint (which was not certified by peer review) is the author/funder, who has granted bioRxiv a license to display the preprint in perpetuity. It is made available under aCC-BY 4.0 International license.

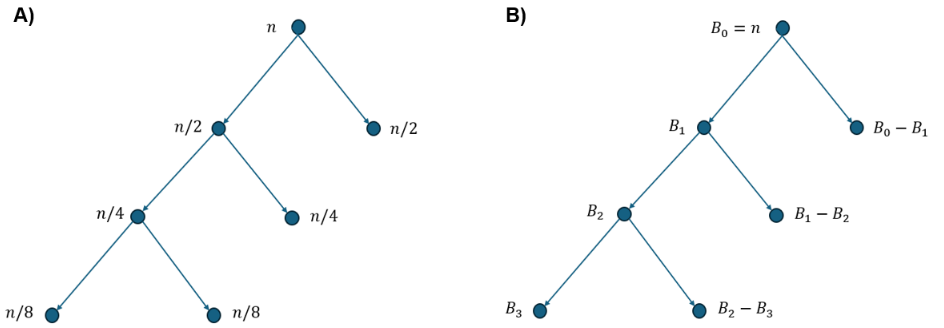

Fig. S4 Illustration of worst-case subtree sizes in random bifurcating tree. A) The worst-case subtree sizes of ancestors (dots) are when each bifurcation is an even split. B) The model $\{B_j\}$ of subtree sizes down one branching path is defined as random variables. The sample size is denoted as n.

bioRxiv preprint doi: https://doi.org/10.1101/2024.12.13.628449; this version posted January 7, 2025. The copyright holder for this preprint (which was not certified by peer review) is the author/funder, who has granted bioRxiv a license to display the preprint in perpetuity. It is made available under aCC-BY 4.0 International license.

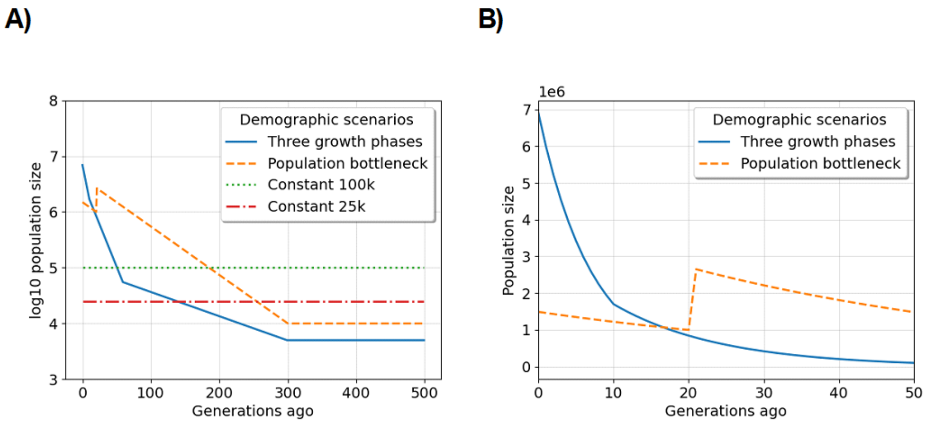

Fig. S5 Demographic scenarios we consider in simulation studies: A) coalescent time in generations ago by the log 10 population size, and B) the most recent fifty generations by population size for examples of exponential growth. The legends specify the color and line style for each scenario. As opposed to coalescent time used in the main text, we describe the scenarios moving forward in time here. The three phases of exponential growth model is as follows: a population of ancestral size five thousand diploids increases exponentially each generation at rates one, seven, and fifteen percent starting three hundred, sixty, and ten generations ago. This demographic model is similar to the "UK-like" model in Cai et al. (2023). The population bottleneck model is as follows: a population of ancestral size ten thousand diploids increases exponentially each generation at a rate of two percent starting three hundred generations ago, but twenty generations before the present day, the population experiences an instantaneous reduction in size to one million diploids. Otherwise, the demographic scenarios we explore here are populations of constant size twenty-five and one hundred thousand diploids.

bioRxiv preprint doi: https://doi.org/10.1101/2024.12.13.628449; this version posted January 7, 2025. The copyright holder for this preprint (which was not certified by peer review) is the author/funder, who has granted bioRxiv a license to display the preprint in perpetuity. It is made available under aCC-BY 4.0 International license.

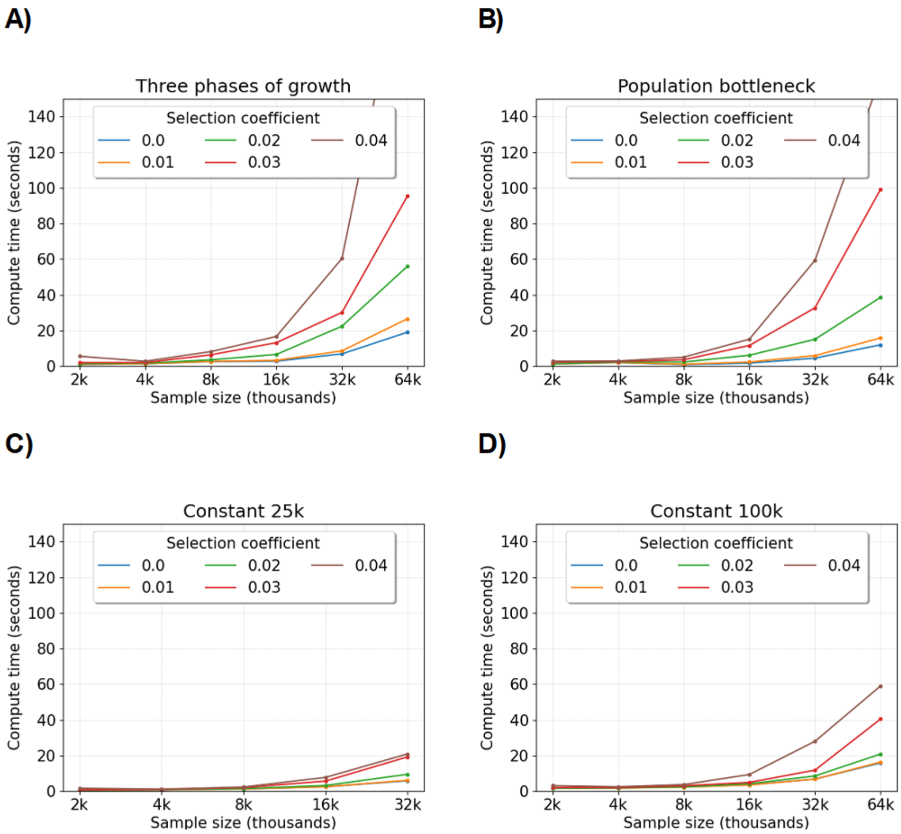

Fig. S6 Compute time to simulate IBD segment lengths around a locus depending on demography and selection. Compute time (y-axis) in seconds by sample size (x-axis) in thousands is averaged over five simulations. The legends denote colored line styles for different selection coefficients. A), B), C), and D) show results for demographic scenarios of three phases of exponential growth, a population bottleneck, and constant population sizes of twenty-five and one hundred thousand diploids, respectively. The Morgans length threshold is 0.01.

bioRxiv preprint doi: https://doi.org/10.1101/2024.12.13.628449; this version posted January 7, 2025. The copyright holder for this preprint (which was not certified by peer review) is the author/funder, who has granted bioRxiv a license to display the preprint in perpetuity. It is made available under aCC-BY 4.0 International license.

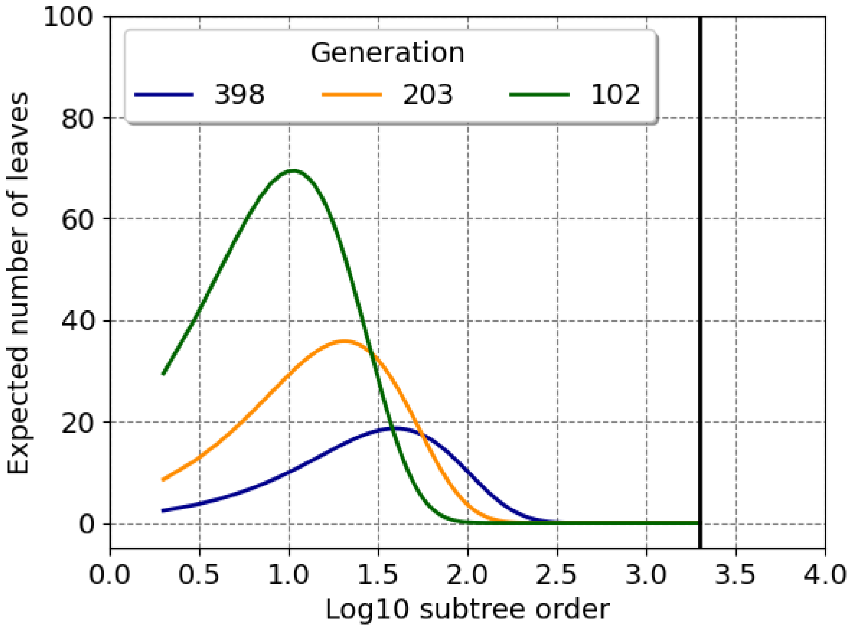

Fig. S7 The expected cardinality of subtree sizes at different coalescent times. Using Lemma 1 in Dahmer and Kersting (2015), we compute the expected number of subtrees containing r samples (x-axis) at the $(n-k)^{\text{th}}$ coalescent event. We multiply these moments by r to get the expected number of leaves under such subtrees (y-axis). There are two thousand samples. Dark blue, orange, and green lines correspond to k =50, 95, and 180. We compute the expected time of the $(n-k)^{\text{th}}$ coalescent event (Hein et al., 2005) and multiply by a population size of ten thousand to get generations (legend). The vertical line is logarithm 10 of sample size.
